# Exceptional Long-Term Survival after Resection of Metachronous Liver and Lung Metastases Following Surgery for Pancreatic Cancer: A Case Report

**DOI:** 10.70352/scrj.cr.26-0115

**Published:** 2026-06-20

**Authors:** Takumi Miwa, Makoto Takahashi, Tatsuya Hayashi, Shogo Shikura, Hayato Sato, Taiki Tsuji, Akinori Ichinose, Taku Higashihara, Yuko Sakaibori, Takuma Kikkawa, Dai Inoue, Hideaki Ishii, Junko Araki, Tomonori Kai, Haruka Okada, Yasuhiro Morita

**Affiliations:** 1 Department of General Surgery, Tokyo Metropolitan Tama Medical Center, Fuchu, Tokyo, Japan; 2 Department of Thoracic Surgery, Tokyo Metropolitan Tama Medical Center, Fuchu, Tokyo, Japan; 3 Department of Gastroenterology and Hepatology, Tokyo Metropolitan Tama Medical Center, Fuchu, Tokyo, Japan; 4 Department of Clinical Genomics, Tokyo Metropolitan Tama Medical Center, Fuchu, Tokyo, Japan; 5 Department of Endocrinology and Metabolism, Tokyo Metropolitan Tama Medical Center, Fuchu, Tokyo, Japan; 6 Department of Radiology, Tokyo Metropolitan Tama Medical Center, Fuchu, Tokyo, Japan; 7 Department of Pathology, Tokyo Metropolitan Tama Medical Center, Fuchu, Tokyo, Japan

**Keywords:** pancreatic ductal adenocarcinoma (PDAC), special AT-rich sequence-binding protein 2 (SATB2), metachronous liver metastasis, metachronous lung metastasis, surgery, chemotherapy, multidisciplinary treatment

## Abstract

**INTRODUCTION:**

Pancreatic ductal adenocarcinoma (PDAC) has a poor prognosis, especially in patients with distant metastases. However, recent advances in multidisciplinary treatment strategies, including surgical resection, have led to successful outcomes even in patients with metastatic PDAC. Nevertheless, few cases have been reported with 2 or more resections of metachronous distant metastases. Herein, we report a case of PDAC in which a metachronous hepatic metastasis was successfully resected following prolonged chemotherapy after curative pancreatectomy and in which a metachronous pulmonary metastasis was subsequently resected following additional chemotherapy.

**CASE PRESENTATION:**

A 69-year-old man was diagnosed with PDAC and received neoadjuvant chemotherapy in October 2019. In March 2020, a subtotal stomach-preserving pancreaticoduodenectomy was performed. The patient received 1 year of adjuvant chemotherapy following surgery. In September 2021, 18 months after surgery, CT showed a 10-mm nodule in segment (S) 6 of the liver, which was diagnosed as a hepatic recurrence of PDAC. After 19 months of chemotherapy, the metastatic liver lesion remained solitary and showed no change in size, suggesting that the tumor was well-controlled. In June 2023, a partial hepatectomy of S6 was performed. Chemotherapy with S-1 was continued after hepatic resection; however, a metastatic pulmonary lesion in the right lower lobe was identified on CT 15 months after the liver surgery. Right lower lobe partial resection was performed using video-assisted thoracoscopic surgery in January 2025. The patient remains alive and recurrence-free 1 year after the last surgery, 6 years and 1 month after the initial surgery, and 6 years and 6 months after the start of treatment.

**CONCLUSIONS:**

This report describes a case of long-term survival achieved with a multidisciplinary approach, including 2 surgical resections, for recurrent disease following a diagnosis of PDAC.

## Abbreviations

*BRCA*
: breast cancer susceptibility genes
CA19-9
: carbohydrate antigen 19-9
CEA
: carcinoembryonic antigen
CK
: cytokeratin
DDR
: DNA damage response
DFI
: disease-free interval
DUPAN-2
: duodenal pancreatic cancer antigen 2
Gd-EOB-MRI
: gadoliniumethoxybenzyl-enhanced MRI
GEM
: gemcitabine
GnP
: gemcitabine plus nab-paclitaxel
IPMN
: intraductal papillary mucinous neoplasm
*KRAS*
: Kirsten rat sarcoma viral oncogene homolog
MDCT
: multi-detector row CT
mFOLFIRINOX
: modified combination of fluorouracil, leucovorin, irinotecan, and oxaliplatin
MUC
: mucin
NAC
: neoadjuvant chemotherapy
PDAC
: pancreatic ductal adenocarcinoma
RECIST
: Response Evaluation Criteria in Solid Tumors
S
: segment
SATB
: special AT-rich sequence-binding protein
SD
: stable disease
SSPPD
: subtotal stomach-preserving pancreaticoduodenectomy
*TP53*
: tumor protein p53
VATS
: video-assisted thoracic surgery

## INTRODUCTION

The prognosis of PDAC is dismal, especially in patients with postoperative recurrence.^1)^ The liver, lungs, and lymph nodes are predominant sites of metastasis.^2)^ Historically, only remnant pancreatectomy was considered a surgical indication for metachronous recurrence of PDAC.^3)^ However, owing to recent advances in multidisciplinary treatment, including chemotherapy and radiotherapy, a few cases of long-term survival have been reported in patients with metachronous metastases of PDAC, notably in the lungs.^4)^ Regarding metachronous liver metastasis, the prognosis is exceptionally poor, and surgeries are rarely performed.^5)^ Furthermore, few reports have documented long-term survival after repeated resections of metachronous metastatic recurrences following PDAC surgery.^6,7)^

Herein, we report a case of long-term survival following multidisciplinary treatment, including surgical resections, for metachronous liver and lung metastases after curative resection of PDAC.

## CASE PRESENTATION

A 69-year-old man with a history of diabetes mellitus was admitted to our hospital for exacerbation of his diabetes and jaundice. MDCT showed a 30-mm nodule at the pancreatic head (**Fig. 1**). Endoscopic retrograde cholangiopancreatography was performed, and a self-expanding metallic stent was placed at the site of bile duct stenosis. Endoscopic ultrasonography was performed, and biopsy of the nodule revealed an adenocarcinoma, which was diagnosed as PDAC. Blood test results were as follows: CA19-9, 1808 U/mL (normal values: <37.0), and CEA, 4.1 ng/mL (normal values: <5.0). In October 2019, 2 courses of GEM plus S-1 were initiated as NAC. GEM was administered at a dose of 1000 mg/m^2^ on days 1 and 8 of a 21-day cycle, whereas S-1 was administered at a dose of 60 mg/m^2^ twice daily on days 1–14. CA19-9 levels decreased to 263.0 U/mL, while DUPAN-2 and CEA levels were 830 U/mL and 2.9 ng/mL, respectively. However, the surgery was postponed due to worsening diabetes, slight tumor enlargement on CT (RECIST: SD), and suspected tumor invasion extending to the root of the gastroduodenal artery. Accordingly, in December 2019, 3 courses of GnP therapy were administered. GEM was administered at a dose of 1000 mg/m^2^ and nab-paclitaxel at a dose of 125 mg/m^2^ on days 1 and 8 of a 21-day cycle.

**Fig. 1:**
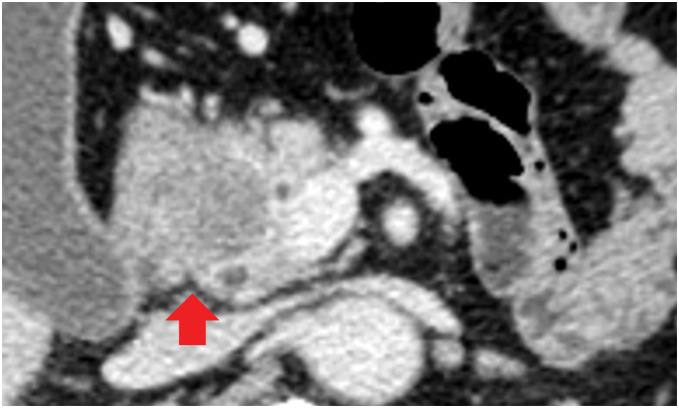
Multi-detector row CT showing a 30-mm hypovascular tumor (red arrow) in the pancreatic head.

After completing 3 cycles of GnP, the levels of CA19-9 had decreased to 78.8 U/mL, DUPAN-2 to 160 U/mL, and the CEA level was 2.7 ng/mL. SSPPD was subsequently performed in March 2020. The operative time was 7 h 23 min, and the estimated blood loss was 487 mL. Postoperatively, delayed gastric emptying was observed; however, the condition improved with conservative management, and the patient was discharged on POD 24. Based on the pathological findings, the tumor was classified as T2N1M0 stage IIB according to the 8th edition of the Union for International Cancer Control classification, with R0 resection (**Fig. 2**). Of the 50 dissected lymph nodes, only 1 posterior pancreaticoduodenal lymph node (station #13, according to the 8th edition of the Japan Pancreas Society classification of pancreatic carcinoma^8)^ showed metastasis. The tumor was well to moderately differentiated with mild lymphatic, venous, and perineural invasion. The histological response to chemotherapy was minimal, corresponding to Evans grade Ia.^9)^

**Fig. 2:**
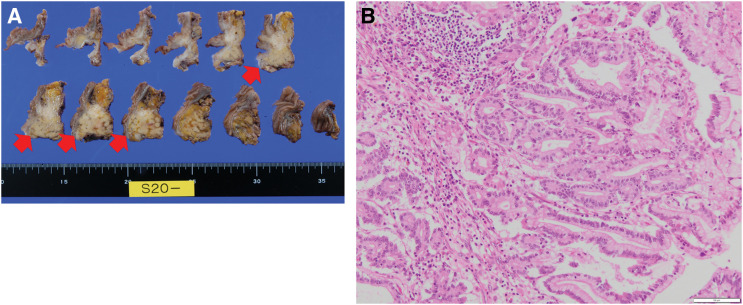
Pathological findings of the 1st surgery, a subtotal stomach-preserving pancreaticoduodenectomy. (**A**) Macroscopic examination of the resected specimen identified a 30-mm nodule in the pancreatic head (red arrows). (**B**) Histopathological findings (hematoxylin–eosin staining) showing invasive ductal adenocarcinoma. Scale bar: 100 μm.

S-1 was then administered for 1 year as adjuvant chemotherapy. However, 1 year and 6 months after surgery, MDCT revealed a 10-mm nodule in S6 of the liver, which was considered a metachronous liver metastasis (**Fig. 3A**); Gd-EOB-MRI was consistent with PDAC metastasis (**Fig. 3B**). Based on these findings, we diagnosed the S6 liver nodule as a metachronous metastasis of PDAC. At that time, the CA19-9 level was 35.3 U/mL, the DUPAN-2 level was ≤25 U/mL, and the CEA level was 1.6 ng/mL.

**Fig. 3:**
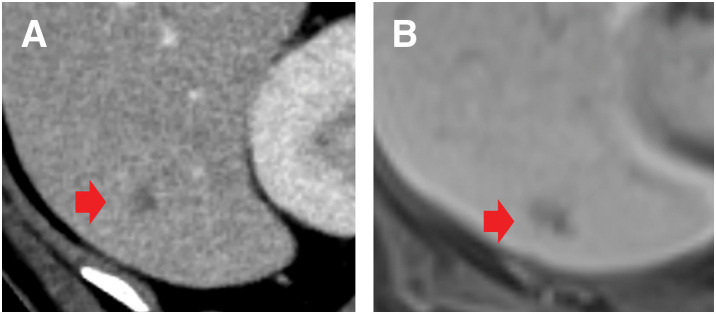
Imaging findings of the hepatic metastasis. (**A**) Multi-detector row CT performed 18 months after pancreaticoduodenectomy showing a 5-mm hypovascular lesion in hepatic segment 6 (red arrow). (**B**) Gadoliniumethoxybenzyl-enhanced MRI showing the lesion with low-signal intensity in the hepatobiliary phase (red arrow).

The patient had no family history of pancreatic or related malignancies. Genetic testing revealed no germline mutations in the *BRCA1* or *BRCA2* or other DDR-related genes. Somatic mutation analysis revealed *KRAS* and *TP53* pathogenic mutations.

In October 2021, 17 courses of mFOLFIRINOX were initiated. Fluorouracil (2400 mg/m^2^), leucovorin (200 mg/m^2^), irinotecan (150 mg/m^2^), and oxaliplatin (85 mg/m^2^) were administered in a 14-day cycle. However, in June 2022, although the liver metastatic lesions were radiologically assessed as SD, the CA19-9 level increased to 103.7 U/mL, the DUPAN-2 level to 75 U/mL, and the CEA level to 6.6 ng/mL. In addition, peripheral neuropathy worsened as an adverse effect, leading to the decision to switch to GnP therapy. Eleven courses of GnP were administered from July 2022 to May 2023. The total treatment duration was 19 months. As of December 2022, the CA19-9 level decreased to 56.6 U/mL, the DUPAN-2 level to 68 U/mL, and the CEA level to 3.6 ng/mL. Thereafter, only the CA19-9 level gradually increased, reaching 78.8 U/mL in May 2023, whereas the DUPAN-2 and CEA levels were 51 U/mL and 3.3 ng/mL, respectively. Over the course of 19 months, the S6 liver lesion showed no change in size, and no new metastatic lesions were identified. After informed consent was obtained, surgical resection was selected as the treatment strategy. In June 2023, 3 years and 3 months after SSPPD, a partial hepatectomy of S6 was performed. No additional intraoperative lesions were observed. The operative time was 3 h 37 min, and the estimated blood loss was 39 mL. The postoperative course was uneventful, and the patient was discharged on POD 5. Pathological examination revealed that the tumor was 12 mm in size and consistent with PDAC metastasis. The resection margins were negative (**Fig. 4**).

**Fig. 4:**
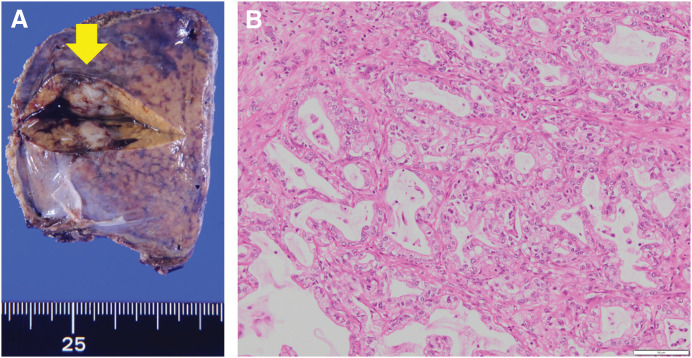
Pathological findings of the 2nd surgery, a partial hepatectomy. (**A**) Macroscopic examination of the resected specimen identified a 12-mm white nodule (yellow arrow). (**B**) Histopathological findings (hematoxylin–eosin staining) showing atypical cells with enlarged, hyperchromatic, oval nuclei and abundant mucin, proliferating and infiltrating in fused glandular and small tubular structures. These findings were similar to those observed in the initial surgical specimen, and the lesion was diagnosed as a hepatic metastasis of pancreatic ductal adenocarcinoma. Scale bar: 100 μm.

Following a discussion with the patient, oral S-1 therapy (80 mg/m^2^) was restarted on POD 21. In September 2024, 4 years and 6 months after SSPPD and 1 year and 3 months after partial hepatectomy of S6, MDCT revealed a small nodule in the right lower lobe of the lungs (**Fig. 5**). As no other lesions were observed and assessing malignancy was difficult, surgical intervention was planned for diagnostic and therapeutic purposes. At that time, CA19-9 was 46.6 U/mL, DUPAN-2 was 68 U/mL, and CEA was 3.4 ng/mL.

**Fig. 5:**
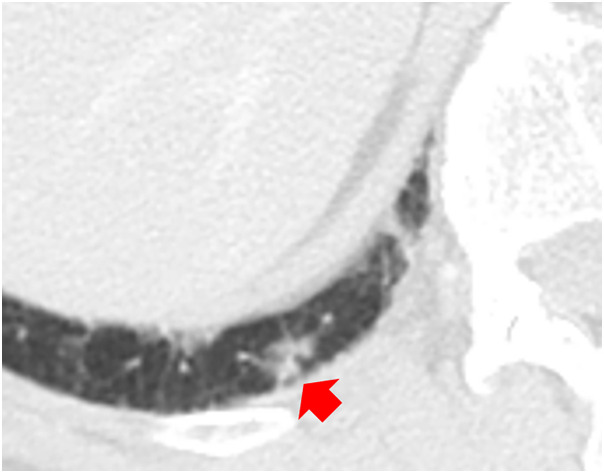
Multi-detector row CT performed 4 years and 6 months after the pancreaticoduodenectomy showing a small nodule in the right lower lobe of the lung (red arrow).

In January 2025, VATS was performed for a right lower lobe partial resection. The operative time was 2 h 5 min, and blood loss was minimal. The postoperative course was uneventful, and the patient was discharged on POD 2. Pathological examination revealed that the tumor was 10 mm in size and consistent with PDAC metastasis. The resection margins were negative (**Fig. 6**). Immunohistochemical analysis showed similar staining profiles across the pancreatic, hepatic, and pulmonary specimens, showing positivity for CK7, MUC1, and SATB2; negativity for CK20 and MUC2; and partial positivity for MUC5AC and MUC6 (**Fig. 7**).

**Fig. 6:**
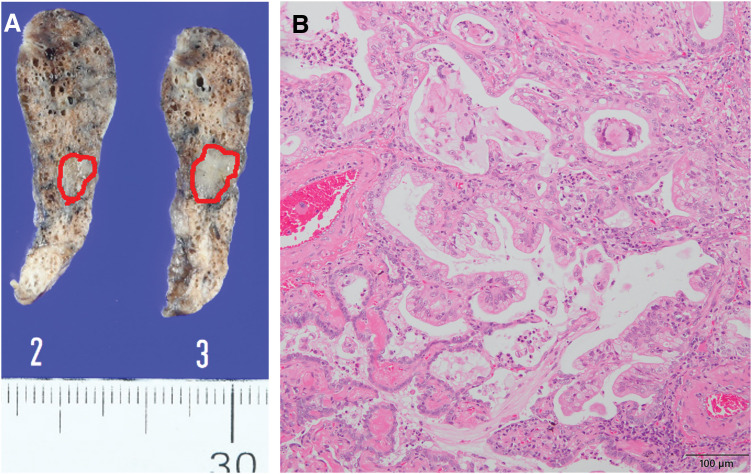
Pathological findings of the 3rd surgery, a right lower lung lobectomy. (**A**) Macroscopic examination of the resected specimen identified a 10-mm gray-white nodule (indicated by the red outlines). (**B**) Histopathological findings (hematoxylin–eosin staining) showing atypical glandular epithelium proliferating in an acinar or papillary pattern with an alveolar-replacing growth. The tumor cells were tall columnar with nuclear enlargement and anisokaryosis; these findings were consistent with metastasis from the primary pancreatic ductal adenocarcinoma. Scale bar: 100 μm.

**Fig. 7:**
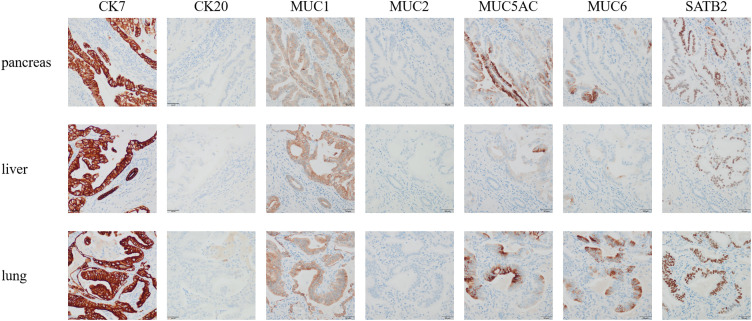
Immunohistochemical findings showing a similar staining profile across the pancreatic, hepatic, and pulmonary specimens, demonstrating positivity for CK7, MUC1, and SATB2; negativity for CK20 and MUC2; and partial positivity for MUC5AC and MUC6. CK, cytokeratin; MUC, mucin; SATB, special AT-rich sequence-binding protein

The patient remains alive and recurrence-free 1 year and 3 months after VATS, 2 years and 10 months after hepatectomy, 6 years and 1 month after SSPPD, and 6 years and 6 months after treatment initiation. The clinical course of this case, including changes in tumor marker levels, is shown in **Fig. 8**.

**Fig. 8:**
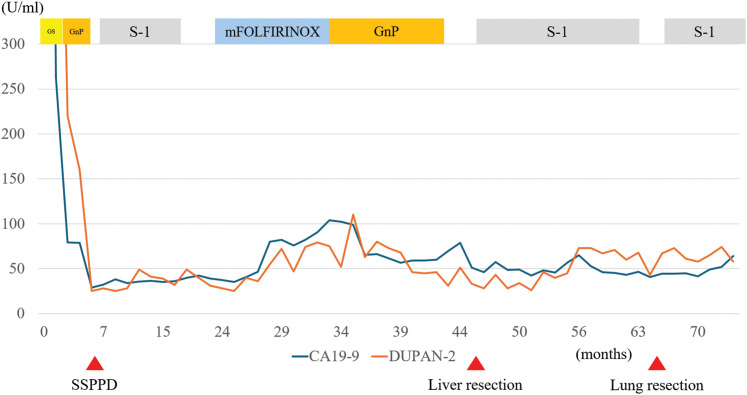
Clinical course of this case. CA19-9, carbohydrate antigen 19-9; DUPAN-2, duodenal pancreatic cancer antigen 2; GnP, gemcitabine plus nab-paclitaxel; GS, gemcitabine plus S-1; mFOLFIRINOX, modified combination of fluorouracil, leucovorin, irinotecan, and oxaliplatin; SSPPD, subtotal stomach-preserving pancreaticoduodenectomy

## DISCUSSION

Despite advances in multimodal treatment, PDAC remains one of the most lethal malignancies, with a 5-year overall survival rate of approximately 10%.^10)^ Prognosis worsens significantly in patients who develop postoperative recurrence, which typically occurs within the first year after primary surgery. The benefits of surgical resection for postoperative PDAC recurrence have been demonstrated in remnant pancreatic recurrence.^3)^ In addition, surgical resection may be considered in cases of pulmonary metastatic recurrence when chemotherapy has been effective for patients with oligometastatic disease that developed late after the initial surgery and remains confined to the lungs.^4,11)^ However, accumulating evidence suggests that surgical management combined with systemic therapy may offer a survival benefit in select patients with metachronous oligometastatic disease, particularly in those involving the liver and lungs.

Liver metastases from PDAC are commonly detected within 1 year after pancreatectomy and often present as multifocal or disseminated disease; consequently, these cases are unsuitable for surgical intervention. Instead, first-line chemotherapy regimens, such as GnP or FOLFIRINOX, are considered standard therapies,^12,13)^ offering modest survival benefits. However, some patients with limited metachronous hepatic metastases demonstrate a favorable response to chemotherapy and prolonged DFI, and these patients may be candidates for hepatic resection.

Several retrospective studies have identified key prognostic factors associated with improved outcomes after surgical intervention for liver metastasis. Mitsuka et al. reported that patients who underwent liver resection had a significantly longer median DFI (21 vs. 3 months in unresectable cases) and fewer metastatic lesions (median, 1 vs. 7), underscoring the importance of tumor biology and burden in patient selection.^14)^ Schwarz et al. similarly emphasized that well-controlled disease, defined by tumor shrinkage and decreased tumor marker levels during chemotherapy, is critical before considering hepatectomy.^5)^

Nakata et al. reported on the resection of synchronous liver metastases from PDAC following prolonged chemotherapy, defined oligometastasis as 3 or fewer lesions, and identified a preoperative CA19-9 level of ≤100 U/mL, a CEA level of ≤5 ng/mL, and resectability status of resectable or borderline resectable disease as factors associated with long-term survival after resection of oligometastatic liver disease. The duration of preoperative chemotherapy ranged from 1.5 to 15 months, with a median of 5.5 months.^15)^

In our patient, only 1 hepatic metastasis was identified 18 months after SSPPD, which is a relatively long interval for PDAC recurrence. Considering the effect of combining systemic therapy with surgical resection in oligometastatic PDAC, partial hepatectomy was performed after 19 months of SD on mFOLFIRINOX and GnP. This approach aligned with the consensus that a DFI longer than 3 months, limited tumor numbers (generally <3), and SD during chemotherapy are favorable indicators for surgery. The duration of preoperative chemotherapy before hepatectomy was 19 months, which was considerably longer than that reported in previous studies. During this period, although the CA19-9 level initially decreased, it subsequently increased, prompting a change in chemotherapy regimen from mFOLFIRINOX to GnP. After GnP administration, CA19-9 levels declined temporarily, followed by a slight increase. As imaging revealed no significant changes in liver metastasis and no new lesions, hepatic resection was performed. The preoperative CA19-9 level was <100 U/mL and the CEA level was <5 ng/mL, meeting the criteria proposed by Nakata et al.^15)^

As for pulmonary metastasis, isolated pulmonary recurrence after PDAC resection is less common but has been associated with a comparatively better prognosis. Zheng et al. demonstrated that patients with primary lung recurrence had significantly improved survival rates compared with those with hepatic or other metastatic sites, possibly because of the distinct tumor biology or slower progression.^16)^ In a nationwide multicenter analysis, Homma et al. confirmed that lung metastases of PDAC often present as solitary lesions and may be amenable to surgical resection, which was associated with prolonged survival.^17)^ In summary, Yamanaka et al. suggested that single-organ metastasis, countable lesions, and lower CA19-9 levels are good indications for surgical resection of oligochronous metastases of PDAC.^18)^

Recently, an increasing number of reports have demonstrated successful treatment outcomes, including resection, of oligometastases following PDAC surgery. However, there are very few reports on patients undergoing 2 or more additional surgeries for metachronous metastases after PDAC surgery. Hagiwara et al. described a patient who underwent hepatectomy followed by pulmonary metastasectomy for a total of 5 surgeries for metastases, achieving 10 years of long-term recurrence-free survival.^7)^ Similarly, Aizawa et al. reported a case of metastatic PDAC treated with chemotherapy followed by resection of liver and pulmonary metastases, resulting in an extended survival of 6 years.^6)^ Together with our case, these reports highlight that aggressive multimodal treatments, including systemic chemotherapy and staged metastasectomy, can offer select patients a chance for prolonged disease control and survival.

Here, we discuss the molecular, biological, and genetic features of this case. In this case, SATB2 positivity was observed in all lesions, including the primary PDAC, as well as in the liver and lung metastases. SATB2 is frequently expressed in colorectal cancer^19)^; however, its expression in PDAC is rare, and some studies have reported that SATB2 expression is not observed in IPMN.^20)^ In the present case, multiple colonoscopic examinations were performed both before and after surgery, and no findings suggestive of colorectal cancer were identified. Furthermore, neither preoperative imaging nor histopathological findings suggested pancreatic cancer arising from an IPMN.

In colorectal cancer, SATB2 expression has been associated with a well-differentiated histology, low malignant potential, and a favorable prognosis.^21)^ In contrast, SATB2-positive cases of PDAC account for only a small proportion of cases, and although their clinical significance remains unclear, some studies have suggested a possible association with poor prognosis.^22,23)^ Despite being SATB2-positive for PDAC, the present case demonstrated a relatively favorable clinical course, which is noteworthy and may be inconsistent with previously reported findings.

Furthermore, from a genetic standpoint, although no germline mutations in *BRCA1*, *BRCA2*, or other DDR-related genes were identified, the presence of *KRAS* and *TP53* pathogenic mutations is consistent with the typical PDAC molecular profile. Therefore, the favorable clinical course observed in this case may not be explained by known DDR-related genomic alterations, suggesting that other factors, such as tumor differentiation or biological behavior, may have contributed to the relatively indolent disease progression.

Although surgery for metastatic PDAC remains controversial and is applicable to only a minority of patients, accumulating evidence supports a paradigm shift toward individualized treatment strategies. The key factors for considering metastasectomy include a prolonged DFI (preferably >12 months), limited numbers of metastatic lesions, favorable response or SD under systemic chemotherapy, and the absence of widespread or extra-organic metastases. In our patient, even though the metastases were found in 2 different organs, they were found in sequential order and not at the same time; furthermore, the metastatic tumors were well controlled with chemotherapy. Nonetheless, balancing the risks and benefits, optimizing the timing of surgery, and integrating systemic therapies remain controversial in patients with metachronous PDAC metastases. Even in cases such as the present case, characterized by elevated tumor markers at the initial visit, lymph node metastasis in the resected specimen, and positive lymphatic, venous, and perineural invasion, indicating high malignancy, as well as no evidence of low tumor aggressiveness from a genetic standpoint, patients who can tolerate long-term chemotherapy and maintain reduced tumor marker levels may achieve favorable outcomes with multimodal treatment, including resection. It is crucial to administer prolonged chemotherapy prior to surgery for metachronous metastases and assess the treatment response to determine whether surgical intervention is truly beneficial. Further prospective studies are needed to refine the selection criteria and evaluate the duration of preoperative therapy before surgical resection.

## CONCLUSIONS

Herein, we report a case in which long-term survival was achieved after curative resection of PDAC by resecting a metachronous hepatic metastasis after prolonged systemic chemotherapy, followed by surgical resection of a subsequently developed pulmonary metastasis. Although the prognosis for postoperative recurrence of PDAC is generally poor, some patients can achieve long-term survival through prolonged systemic chemotherapy combined with repeated surgical resections.

## DECLARATIONS

### Availability of data and materials

The dataset supporting the conclusions of this article is included within the article.
